# Swine as the Animal Model for Testing New Formulations of Anti-Inflammatory Drugs: Carprofen Pharmacokinetics and Bioavailability of the Intramuscular Route

**DOI:** 10.3390/pharmaceutics14051045

**Published:** 2022-05-12

**Authors:** Lidia Gómez-Segura, Antoni Boix-Montañes, Mireia Mallandrich, Alexander Parra-Coca, José L. Soriano-Ruiz, Ana Cristina Calpena, Álvaro Gimeno, David Bellido, Helena Colom

**Affiliations:** 1 Department of Medicine and Animal Health, Faculty of Veterinary, Autonomous University of Barcelona, 08193 Bellaterra, Spain; lidia.gose@gmail.com; 2 Department of Pharmacy and Pharmaceutical Technology and Physical Chemistry, Faculty of Pharmacy and Food Sciences, University of Barcelona, 08028 Barcelona, Spain; antoniboix@ub.edu (A.B.-M.); anacalpena@ub.edu (A.C.C.); helena.colom@ub.edu (H.C.); 3 Institute of Nanoscience and Nanotechnology (IN2UB), University of Barcelona, 08028 Barcelona, Spain; 4 Department of Veterinary Medicine and Zootechnic, Faculty of Agriculture Sciences, University of Applied and Environmental Sciences, Bogota 111166, Colombia; aleparra@udca.edu.co; 5 Department of Pharmacy and Pharmaceutical Technology, Faculty of Pharmacy, University of Granada, 18071 Granada, Spain; jlsoriano@correo.ugr.es; 6 Department of Animal Research, Animal House of Bellvitge, University of Barcelona, CCiT-UB, 08907 L’Hospitalet de Llobregat, Spain; alvarogimeno@ub.edu; 7 Department of Separative Techniques, Scientific and Technological Centers, University of Barcelona, 08028 Barcelona, Spain; bellido@ccit.ub.edu

**Keywords:** population pharmacokinetics, tissue distribution, swine animal model, mass spectrometry, bioanalysis, carprofen, anti-inflammatory drugs

## Abstract

Carprofen (CP) is a non-steroidal anti-inflammatory drug (NSAID) frequently used to treat respiratory diseases in numerous small animals, but also in large species. CP is a formidable candidate for further therapeutic research of human inflammatory diseases using the pig as an animal model. However, CP administration in swine is very uncommon and respective pharmacokinetics/bioavailability studies are scarce. A simultaneous population pharmacokinetic analysis after CP intravenous and intramuscular administrations in pigs has shown high extent and rate of absorption and a similar distribution profile with respect to man and other mammals. However, clearance and half-life values found in swine suggest a slower elimination process than that observed in man and some other animal species. Although not reported in other species, liver and kidney concentrations achieved at 48 h post-intramuscular administration in pigs were ten times lower than those found in plasma. Simulations pointed to 4 mg/kg every 24 h as the best dosage regimen to achieve similar therapeutic levels to those observed in other animal species. All these findings support the use of pig as an animal model to study the anti-inflammatory effects of CP in humans.

## 1. Introduction

Carprofen (CP), 6-chloro-alpha-methyl-9H-carbazole-2-acetic acid, is a non-steroidal anti-inflammatory drug (NSAID) with anti-inflammatory, analgesic and anti-pyretic pharmacological effects [1,2].

Since it was licensed for veterinary use in the 1990s [3], CP has been one of the most widely used NSAIDs in veterinary medicine, with a mechanism of action based on the inhibition of the cyclooxygenase (COX) pathway [4]. The effectiveness of CP has been described in numerous pathologies and species such as osteoarthritis in dogs [5], bovine respiratory diseases (in combination with antibiotics) [6,7], bovine mastitis [8,9,10] as well as anti-inflammatory in dogs or cats, and as analgesic in dogs [11]. CP has also been studied in humans with inflammatory diseases [12,13,14]. Even recent in vitro studies have shown that CP can inhibit a key enzyme in the replication and transcription of the virus responsible for SARS-COVID-19, i.e., the main protease of the virus (M-pro) [15]. This therapeutic usefulness makes this drug a formidable candidate for further investigation, with high scientific attractiveness.

Large animal models such as non-human primates are more reliable models than small animals to replicate human disease pathogenesis as they are physiologically, immunologically and genetically more closely related to humans [16,17], but in contrast, non-human primate studies are not easy to handle. Pharmacokinetic studies are crucial to predict drug exposure to the action site. They are also the first step towards establishing pharmacokinetic–pharmacodynamic relationships using modeling approaches that can be useful to optimize the dosage regimen for each scenario.

The swine model has been used in pharmacokinetic (PK) studies of different drugs to further predict human PK through allometric models. Numerous CP pharmacokinetic studies have been conducted in various species: horses [18,19], donkeys [20], calves [21,22], sheep [23], rabbits [24,25], vultures [26], humans [27], dogs [28,29] and cats [30,31]; however, very few CP PK studies have been published in swine [32], probably because its use in this specie is uncommon. Moreover, CP have never been administered by the intramuscular route in this species. One of the limitations of the PK studies is a low number of animals would reduce the statistical power. In line with this, population PK approaches are a good alternative to individual analysis, since they reduce the inflated interindividual variability associated with the PK parameters in the two-step approaches, leading to robust results. Moreover, this allows reduction of animal resources according to the 3R strategy.

The robustness of the results is also dependent on the applied analytical methods. Concerning drug analysis, the majority of published pharmacokinetic studies have determined CP using high-performance liquid chromatography (HPLC)-UV detection in accordance with methods previously reported [18]. This technique is not sensitive enough for accurate/precise quantification of low CP plasma concentrations [18]. This supports the use of alternative analytical methods such as liquid chromatography coupled with tandem mass spectrometry (LC-MS/MS), which provides several analytical advantages [33] in terms of significant accuracy and precision, and it also reduces plasma contamination and solvent consumption [34,35] when compared with HPLC-UV. Additionally, few analytical methods for CP determination of the racemic in plasma samples have been reported in the literature. Amstrong et al. [18] determined plasma profiles of the racemate in horses, quantifying concentrations up to about 0.05 mg/L. This was a higher concentration than the limit of quantification found in our study (0.064 µg/L) and supports the use of the newly developed and validated analytical technique to quantify CP plasma concentrations in vivo. Iwakawa et al., determined CP in urine and plasma by HPLC [36] and Jedziniak et al. determined CP by Liquid Chromatography-Tandem Mass Spectrometry in milk [35].

Thus, taking into consideration all factors mentioned above, the primary aim of this study is to use the swine as an animal model for testing CP (Rimadyl^®^) as an anti-inflammatory drug model by characterizing the racemic CP pharmacokinetics and bioavailability after its intravenous (IV) and intramuscular (IM) administrations through a population approach. Since this study reports the administration of Rimadyl^®^ for the first time in this species, the secondary objective is to develop and validate a new LC-MS/MS bioanalytical procedure to quantify CP in plasma samples of swine that are more sensitive than those previously reported in the literature.

## 2. Materials and Methods

### 2.1. Animals and Chemicals

Yorkshire-landrace swine (n = 4, 2 males and 2 females) of 25–50 kg were obtained from the animal facility of Bellvitge (University of Barcelona, Barcelona, Spain).

CP (Rimadyl^®^) used for in vivo studies was obtained from Zoetis (Madrid, Spain). Midazolam (Midazolam Gen^®^) and xylazine (Rom-pun^®^) from Bayer Hispania (San Joan Despi, Spain). Ketamine (Imalgene^®^) was purchased from Boehringer Ingelheim (Sant Cugat del Vallès, Spain). Anesthetics used in this study were propofol (Propofol Lipuro^®^ 1%) and thiopental 1 g from B. Braun VetCare (Rubí, Spain) and isoflurane (Forane^®^ 2%) from Centauro Veterinary (Masies de Roda, Spain). Double-distilled water was obtained from a MilliQ^®^ Plus System (Merck Life Science, Madrid, Spain) lab supplied. All other reagents were of analytical grade and supplied by Sigma Aldrich (Madrid, Spain), unless otherwise specified, and used without further purification.

### 2.2. Experimental Design

This study was conducted according to the guidelines of the Declaration of Helsinki and approved by the Animal Experimentation Ethics Committee of the University of Barcelona (protocol code 10617 on 14 June 2019).

Animals were treated with a standard sedation protocol based on IM midazolam (0.17 mg/kg), IM xylazine (2.5 mg/kg) and IM ketamine (3 mg/kg). Then, anesthesia was induced with IV propofol (2.5 mg/kg). Maintenance was achieved with inhaled isoflurane administered by tracheal intubation with a low-pressure balloon under veterinary supervision for 8 h. Then, for animal welfare reasons, the pigs recovered. The last two samples (24 and 48 h post-administration) were extracted with light sedation using IM xylazine (2.5 mg/kg). The femoral artery was then catheterized by a 22G catheter.

The study was carried out according to a crossover design. Firstly, all the animals were given CP (50 mg/mL) intravenously (IV) in the first period, and then intramuscularly (IM) in the second period. In both cases, doses of 4 mg/kg of CP were administered (habitual doses in several animal species), and blood samples were collected before administration (serving as blank plasma) and at 0.5 min, 15 min, 30 min, 1 h, 2 h, 4 h, 6 h, 8 h and 24 h post-administration, but one more blood sample was collected at 48 h after IM administration. A wash-out period of 7 days was left between both administrations to assure that no residual drug remained from the first period.

Blood samples were stored in EDTA tubes and then centrifuged at 3200 rpm for 15 min at 4 °C. The supernatant (plasma) was removed and frozen at −80 °C for further analysis.

At the end of the study, the animals were euthanized by sodium thiopental overdose (1 g). Then, liver and kidney samples were removed by a veterinary surgeon and frozen at −80 °C for further recovery analysis.

### 2.3. Liquid Chromatography-Mass Spectrometry Analysis

#### 2.3.1. Standard Curve

A standard curve was prepared for each day of CP analysis. The standard curve was constructed from 1 to 1000 ng/mL of CP (Capot Chemical, Hangzhou, China) and Carprofen-d3 (Sigma-Aldrich, Madrid, Spain) was added as Internal Standard (IS). The procedure consisted of weighing 10 mg of CP, adding 5 mL of DMSO and 5mL of methanol (stock solution). From this stock solution (1000 mg/mL), different dilutions were made with methanol to the concentrations described above (from 1 to 1000 ng/mL). Subsequently, IS (Carprofen-d3) at 500 ng/mL was added at each point on the standard line.

#### 2.3.2. LC-MS/MS Method

The equipment used was a UPLC Acquity autosampler and binary pump (Waters Co., Milford, MA, USA) coupled to an API 3000^®^ LC/MS/MS system triple quadrupole mass spectrometer (AB Sciex LLC, Framingham, MA, USA). An Acquity UPLC BEH C18 1.7 µm 2.1 × 50 mm column was used as a stationary phase. The column temperature was set at 40 °C. The mobile phase consisted of (A) ammonium format 10 mM at pH 5.00 in MilliQ^®^ water, and (B) methanol: acetonitrile: formic acid (80:20:0.1). The gradient elution profile was chosen as follows: from 0 to 0.1 min, 98% A; from 0.1 min to 1 min, decreased from 98 to 2%, kept at 2% until min 2, and returning to initial conditions at min 2.1 until min 4. The flow was set at 0.35 mL/min.

CP was detected in the negative electrospray ionization (ESI) mode. The ion spray voltage was set at −4500 V and the source temperature at 450 °C. Data were acquired in multiple reaction monitoring (MRM) mode. Transitions selected for CP were 271.8/228.0 for quantization and 271.8/225.9 for confirmation. IS (carprofen-d3) transition was 275.0/231.2.

#### 2.3.3. Analytical Validation Assays

The LC/MS/MS method for the quantification of CP in plasma samples was validated according to recommendations of Guidance for Industry [37,38] and ICH Q2(R1) [39]. Standard curves were made in quadruplicated. From 1 ppb (ng/mL) to 1000 ppb in DMSO/methanol, these comprised seven different points. IS was 500 ppb of carprofen-d3. Validation was performed for two transitions: T_1_ is for transition 271.8/228.0 and T_2_ is for transition 271.8/225.9, to confirm the quantification of the method.

For linearity and range, four independent sets of CP standard solutions were prepared to build calibration curves and injected, one each, on four consecutive days. The tested range was from 1 ppb to 1000 ppb, IS at 500 ppb in standard solutions in all samples. The linearity was evaluated by linear regression and the criterion of acceptance was set at R^2^ > 0.99.

The precision of the method was examined as repeatability. The 10 ppb, 250 ppb (parts per billion) and 750 ppb CP solutions (IS 500 ppb) were injected six times to test the repeatability of the method. Values of mean, standard deviation (SD) and relative standard deviation (RSD) were then calculated (concentrations in ng/mL).

Accuracy was measured from the linearity of the data. The recorded data points were interpolated in the straight-line calibration curve to study the deviation of the recovery values as a measure of accuracy.

Sensitivity given by the limits of detection (LOD) and limit of quantification (LOQ) of the method was evaluated from the signal-to-noise ratio. The LOD was defined as the lowest concentration level resulting in a peak height of three times the baseline noise. The LOQ was defined as the lowest concentration level that provided a peak height with a signal-to-noise ratio of 10. LOD and LOQ were calculated from the 1 ppb dabigatran chromatogram, resulting in values of 3 and 10 ppb, respectively. Specificity was determined by a white plasma sample without IS to rule out possible matrix effect.

The stability of a spiked sample was evaluated in the storage conditions (−80 °C) for one month.

#### 2.3.4. Sample Preparation

Next, 200 µL plasma samples were transferred in 1.5 mL Eppendorf tubes. Subsequently, 25 µL of IS (carprofen-d3, 10 µg/mL) and 475 µL methanol were added and then mixed and centrifuged at 10,000 rpm for 5 min at 4 °C. The supernatant was collected and kept at −80 °C until analysis. Blank samples pre-administration (blank plasma) underwent the same procedure.

### 2.4. Recovery from Liver and Kidney

Liver and kidney samples were obtained after animals were euthanized and the tissues were superficially cleaned with gauze soaked in a 0.05% solution of dodecyl sulphate followed by MiliQ^®^ water. Then, samples were carefully cut into small pieces and accurately weighed. Samples were homogenized with an IKA^®^ ultra Turrax^®^ disperser tube (DT-20 dispersing tube with rotor-stator element, 25/cs, Sigma-Aldrich, Madrid, Spain), and the CP content was extracted with phosphate-buffered solution, pH 7.4, under sonication for 20 min in an ultrasonic bath protected from light. The supernatant was collected and centrifuged at 10000 rpm for 5 min at 4 °C. The resulting extracted solutions were measured using the LC-MS/MS method previously described.

### 2.5. Statistical Analysis

Non-parametric U-Mann–Whitney-test was performed by using the Prism^®^ software, v. 5.01 (GraphPad Software Inc., San Diego, CA, USA) to compare CP recovery in tissues. The statistical significance was set at *p* < 0.05.

### 2.6. Pharmacokinetic Analysis

#### 2.6.1. Non-Compartmental Pharmacokinetic Analysis

The individual plasma concentration–time profiles of CP found after IV and IM administration were analyzed through a non-compartmental analysis (NCA) with Phoenix-64 (Build 8.1.0.3530, Certara, Princeton, NJ, USA) [40]. No concentrations below the limit of quantification of the analytical method were found. Peak plasma concentration (C_max_) and time to peak plasma concentration (T_max_) were determined directly from the pharmacokinetic profiles. The apparent elimination rate constant (λ_z_), was calculated from the terminal slope of the semi-logarithmic concentration–time curve. The apparent elimination half-life (t_1/2z_) was calculated as t_1/2λz_ = 0.693/λ_z_. The area under the plasma concentration–time curve from time zero to the last experimental time (t) with drug concentrations above the limit of quantification (AUC_t_), was calculated by the linear-log trapezoidal rule. The area from time zero to infinity (AUC) was calculated by adding to the AUC_t_ value; the extrapolated area was calculated as the ratio between the predicted concentration (C_t_) at the last sampling time with concentrations above the limit of quantification and λ_z_. The mean residence time (MRT) was given by the ratio of the area under the first moment curve (AUMC) to the area under the zero-moment curve (AUC). In addition to the usual PK parameters (C_max_, T_max_, AUC, λ_z_ and t_1/2λz_ and MRT) the Cmax/AUC ratio representative of the absorption rate was also reported.

The steady-state distribution volume (Vss) and the IM distribution volume associated with the terminal phase of the plasma concentration–time curve (Vdarea) was also calculated (Vss = MRT·CL and Vdarea = D/AUC·λ_z_).

The CP absolute bioavailability was calculated from the ratio of normalized by dose AUC values obtained after the IM and IV administrations.

#### 2.6.2. Pharmacokinetic Modeling Using the Population Approach

The population pharmacokinetic (PPK) analysis was performed with the non-linear mixed-effects modeling (NONMEM) software, version 7.4 (ICON Development Solutions, Ellicott City, MD, USA) [41]. The stochastic approximation expectation-maximization method (SAEM) followed by an important sampling method (IMP) for standard errors and objective function evaluation were used throughout the model-building process. Graphical diagnostics were guided using Xpose version 4.2.1 [42] implemented into R version 3.6.0. and Perl speaks-NONMEM Toolkit (PsN) version 4.7.0 [43] was used for the bootstrap method. All concentration–time data, after IV and IM administrations, were simultaneously analyzed.

#### 2.6.3. Model Development

Firstly, one and two-compartment models with linear elimination and first-order kinetics absorption without or with lag time were tested. The models were parameterized in terms of absorption-rate constant (Ka), distribution clearance (CL_D_), apparent volumes of distribution (V), elimination clearance (CL) and bioavailability (F).

Inter-individual variability (IIV) was evaluated for each PK parameter and modeled exponentially, assuming a log-normal distribution. Linear MU-referencing modeling was applied to take advantage of the EM method. Early in the analysis, complete omega blocks were used, and they were simplified only when necessary. In the case of simplification, the omega values out of the block were fixed to 0.0225 (coefficient of variation of 15%) to allow the EM algorithm to efficiently move the PK parameter while retaining the original intent that all subjects had similar, although not identical, PK parameter values. Additive, proportional and combined (additive + proportional) models were compared to describe the residual error (RE) in drug concentrations. To statistically distinguish between nested models, the difference in the MOFV (−2 × log likelihood) was used because this difference is approximately χ^2^ distributed. A significance level of *p* < 0.005 equivalent to a difference in MOFV of 7.879 for 1 degree of freedom was considered. For non-hierarchical models, the most parsimonious model with the lowest objective function according to the Akaike information criterion (AIC) was considered [44].

The influence of the bodyweight on the pharmacokinetic parameters was investigated. Specifically, a power relationship was used to test the bodyweight effect by either estimating the exponent or fixing it according to the allometry laws as proposed previously [45]. In the last case, the bodyweight exponents were fixed to 0.75 for all flow parameters (plasma clearance (CL) and distribution clearance (CL_D_)) and to 1 for all volume parameters (central and peripheral compartment distribution volumes (Vc and V_P_), standardized to a 70 kg patient. Statistical and clinical relevance of results of both approaches (estimated or fixed allometric exponents) were considered for model selection.

The decrease in the minimum objective function value (MOFV), parameter precision expressed as relative standard error (RSE%), model completion status (e.g., successful convergence or termination), η- and ε-shrinkage values [46], condition number estimated from the ratio of the major to the minor eigenvalue and visual inspection of goodness-of-fit plots with Xpose were also considered for model selection. Goodness-of-fit plots examined for each model included typical population model-predicted (PRED) and individual model-predicted versus observed concentrations or plots of individual weighted residuals (IWRES) and conditional weighted residuals (CWRES) versus time. The randomness around the identity line of observed concentrations versus population (PRED) and individual (IPRED) predicted concentration plots was examined. Likely plots of individual weighted residuals (IWRES) and conditional weighted residuals (CWRES) versus time were evaluated for randomness around zero.

#### 2.6.4. Model Evaluation

Visual predictive checks [47] based on 1000 simulated replicates of the original datasets were constructed to ensure that the simulations from the model reproduced the observed data. Median, 97.5th and 2.5th percentiles of the observations as well as the 97.5% confidence intervals for the median, the 2.5th and 97.5th percentiles of the simulated profiles were compared.

#### 2.6.5. Simulations and Model Applicability

From the final model, three different scenarios were simulated to find the best dosage regimen of CP, i.e., practical, easy to administer and safe for the treatment of housed swine. CP plasma concentration–time profiles after 4 mg/kg single-dose IV and IM administrations every 8, 12 or 24 h were simulated. The best regimen was chosen based on the concentrations considered therapeutic in other species (15–20 µg/mL) [27,28].

## 3. Results

### 3.1. Liquid Chromatography-Mass Spectrometry

Several ionograms of each swine and blank sample chromatograms can be consulted in the Supplementary Material (Figures S1–S5). Retention times were in the vicinity of 1.51 min approximately.

It could be verified by the two transitions that the values of R^2^ > 0.99. As the RSD of six measurements was better than 3%, the repeatability of the method was considered good. Obtained results are shown in Table 1.

Values of accuracy were from 80 to 120% (see Table 2). Finally, LOD T_1_ was 0.019 ppb and LOQ T_1_ 0.064 ppb. Equally, LOD T_2_ was 0.060 ppb and LOQ T_2_ 0.199 ppb. Ionograms of LOD and LOQ for both transitions T_1_ and T_2_ can be consulted in the Supplementary Material (Figure S6). No interference or matrix effect was observed in the analyzed sample of black plasma (Figures S7 and S8). No changes were observed in spiked samples kept at −80 °C for one month. Based on these results, the samples were stable at the storage conditions for at least one month.

### 3.2. Recovery from Liver and Kidney

The median (min, max) CP recovery values in the liver and kidney after IM administration are shown in Table 3. According to these results, higher CP concentrations remained in the kidney than in the liver at 48 h post-administration, although no statistically significant differences were found.

### 3.3. Carprofen Plasma/Tissue Concentrations

**Figure 1:**
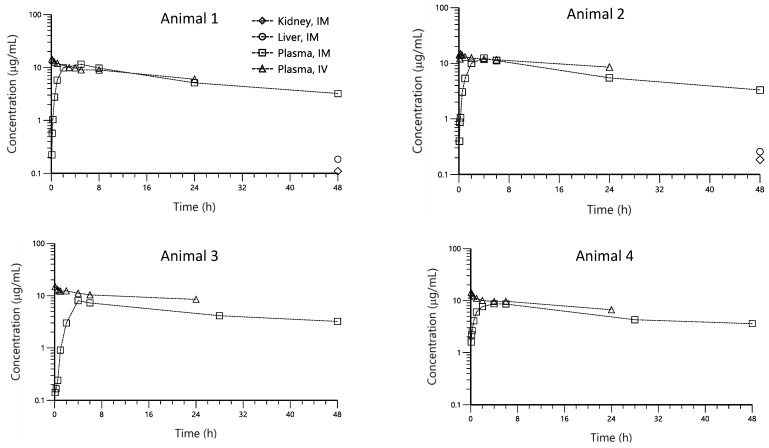
Overlayed individual plots of CP plasma concentration (µg/mL) vs. time (hr) profiles observed following intravenous (Δ) and intra-muscular (☐) administration at the dose of 4 mg/kg in swine, sorted by the animal. CP kidney (◊) and liver (○) concentrations at 48 h are also displayed for animals 1 and 2.

The individual plasma pharmacokinetic profiles after IV and IM administrations sorted by animal are displayed in Figure 1. On the same plots, liver and kidney concentration values achieved at 48 h post-administration, when available (animals 1 and 2), are displayed. Comparatively lower CP concentrations were found in both tissues with respect to plasma.

After IV administration, CP showed a two-exponential decay, with a very long terminal phase accounting for most of the area under the plasma concentration–time profile.

After IM administration, a relatively rapid absorption process occurred until the peak and then a parallel decay to the IV profile took place. This should be expected after the IM administration for a drug with no permeability limitations such as CP, given as a solution. Since no flip-flop took place, the lack of data beyond the 24 h post-IV administration was not a limitation for an adequate characterization of the terminal disposition phase because this information was provided by the IM administration in the simultaneous analysis of all data (IM and IV) as described in the population analysis section. On the other hand, the visual inspection of plots displayed in Figure 1, suggest that IM administration provides high CP bioavailability.

### 3.4. Carprofen Pharmacokinetic Analysis

#### 3.4.1. Non-Compartmental Analysis

Table 4 summarizes the mean values of CP disposition and absorption parameters estimated by the non-compartmental approach. According to these results, high extrapolated areas were found due to insufficient sampling during the terminal phase covering less than three half-lives, especially after the IV route. This is a limitation when a non-compartmental approach is applied for PK parameter estimation. However, this was not a limitation for this study, because as mentioned before, a population pharmacokinetic analysis was performed and data from both administration routes were simultaneously analyzed, providing an adequate description of CP PK. A comparative table including pharmacokinetic parameter values after IV administration to different animal species including swine is displayed in the Supplementary Material (Table S1).

#### 3.4.2. Population Pharmacokinetic Analysis

A total of 73 CP plasma concentrations were analyzed by the population approach, 39 determined after IM and 34 determined after IV administrations. There were no concentrations below the limit of quantification. A two-compartment model with first-order absorption and linear elimination from the central compartment best described CP plasma concentrations. Lag time was not statistically significant in the model. Inter-individual variability (IIV) could be associated with plasma clearance, central distribution volume and absorption-rate constant. The IIV of the remaining PK parameters was fixed to 15%. The inclusion of the off-diagonal elements of the variance–covariance matrix for random effects of Vc, CL and Ka led to instability, increasing the condition number, so it was not retained in the model. The inclusion of bodyweight was not supported by the data, so this covariate was removed from the model. This could be due to the low sample size included in the analysis. Although a wide range of bodyweight values existed, only the bodyweight of one animal deviated about 50% from the mean value.

**Figure 2:**
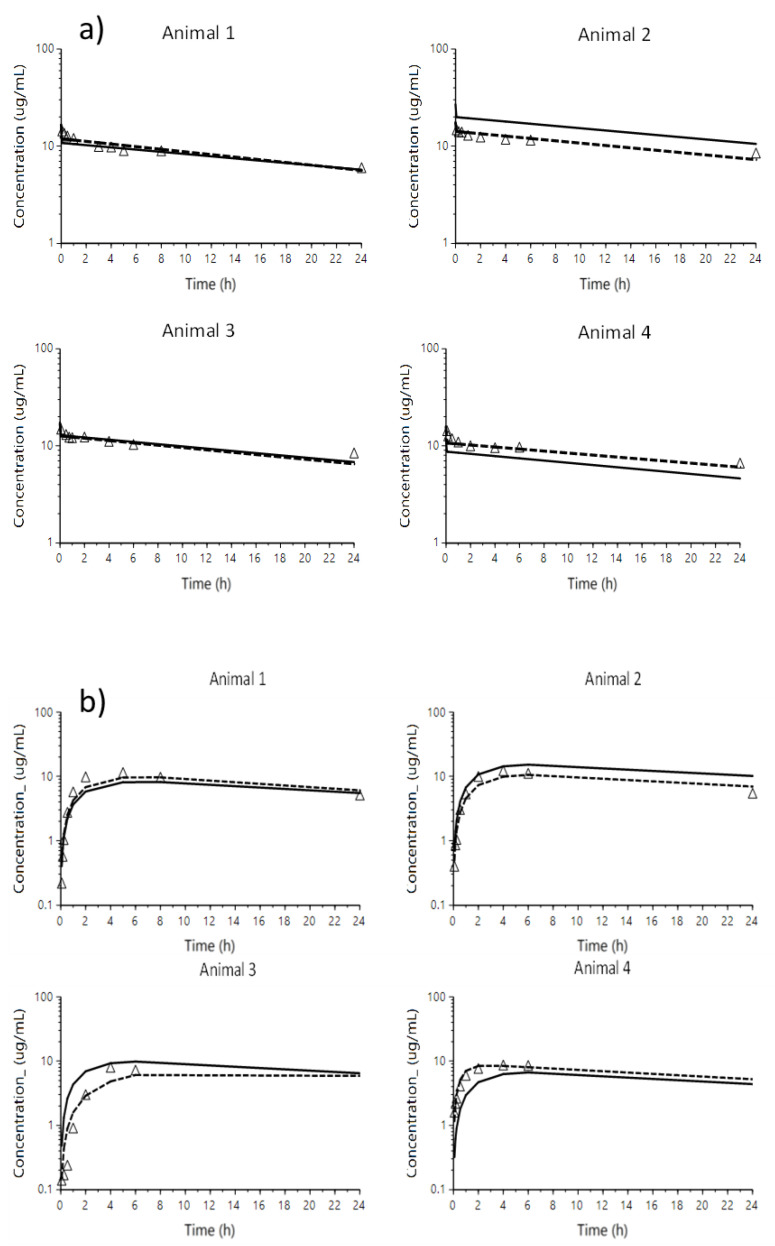
Plots of overlayed observed (triangles), individual predicted (dashed line) and population (solid line) predicted concentrations by the model vs. time profiles, sorted by animal after IV ((**a**) panel) and IM ((**b**) panel) administrations.

**Figure 3:**
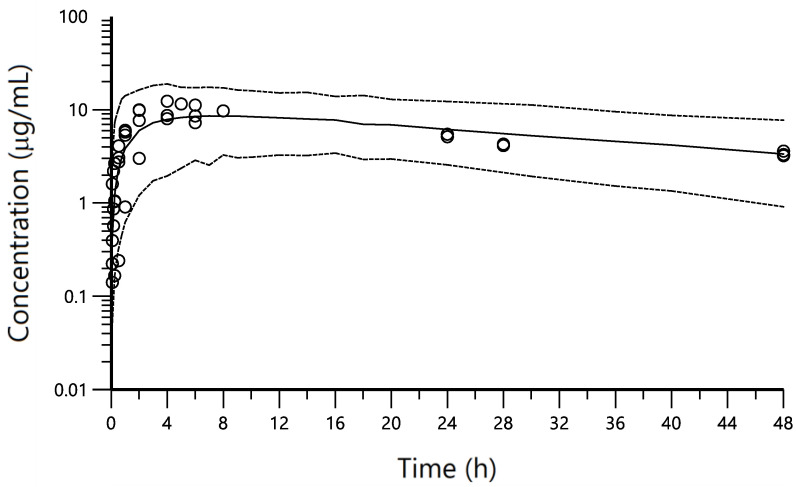
Predictive check of the pharmacokinetic model for CP after the IV and IM administrations. The VPCs were constructed from the fixed and random estimates obtained from the final selected model. One thousand concentration–time profiles were simulated using Monte Carlo simulations after each administration route and their non-parametric 95% confidence intervals (the 2.5th and 97.5th percentiles) were calculated and represented together with the observed data for visual inspection. The circles represent the observed data. Dashed lines depict the 2.5th and 97.5th percentiles of the simulated concentrations. The solid line corresponds to the 50th percentiles of the simulated concentrations. VPC showed that most of the data fell within the 90% prediction interval and were symmetrically distributed around the median both after IV and IM administrations.

Residual variability was best described by a proportional error model. The condition number of this model was 85.53, suggesting no notable collinearity. The final population pharmacokinetic parameters are summarized in Table 5. All the pharmacokinetic parameters were estimated with acceptable precision except IIV associated with Ka. Shrinkages to the mean of IIVCL, IIV Vc and IIV Ka as of ε were of 13.2 (ηCL), 7.29 (ηVc), 1.91 (ηKa) and 8.4% (ε). The goodness-of-fit plots (Figure S9) suggested that in general, the data are well described by the model. However, predicted concentrations of animal 2 were higher than those observed, especially after IV administration (Figure 2). This could be due to the limitation of not having been able to enter the bodyweight in the model. Indeed, this animal showed about 50% higher bodyweight compared to the mean value of the sample. Overall, the VPC (Figure 3) suggested that the model can adequately predict the PK profile of observed CP concentrations. The estimated median represented the trend of the observed data well. In addition, the majority of the observed concentrations fell within the 95% prediction interval, indicating that the predicted variability did not exceed the observed variability.

#### 3.4.3. Simulations and Model Applicability

**Figure 4:**
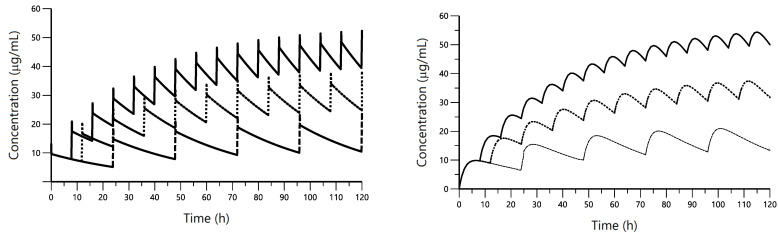
Plots of overlayed simulated concentrations achieved after IV (**left**) and IM (**right**) administration of CP at 3 and 4 mg/kg, respectively, every 8 (solid line), 12 (dotted line) and 24 h (dashed line).

Figure 4 shows the mean pharmacokinetic profiles achieved after CP administration at the dose of 3 mg/kg and 4 mg/kg every 8, 12 and 24 h intravenously and intramuscularly, respectively. According to these results, and taking into account the CP concentrations considered therapeutic in humans and dogs (15–20 µg/mL) [27,28], the best dose regimen for pigs would be the administration of 4 mg/kg every 24 h intramuscularly and 3 mg/kg every 24 h intravenously.

## 4. Discussion

To the best of our knowledge, this is the first study that determined the IM bioavailability of CP at the dose of 4 mg/kg in anesthetized swine, which has become a useful animal model in the research of human diseases. This required previous CP disposition characterization after IV administration at the same dose.

The use of population pharmacokinetic simultaneous analysis of all data from both administration routes allowed a correct evaluation of the CP pharmacokinetics, despite the low number of animals included in the study. Unlike the classical two-step approach, where individual pharmacokinetic analysis followed by descriptive statistics is applied, one-step analysis through the non-linear mixed models allowed reduction of the inflation of between-individual variability associated with pharmacokinetic parameters. Results of the modeling approach were in agreement with those of the non-compartmental analysis that confirmed the descriptive capability of the developed model.

As reported by Bošnjak M. et al. [32], the CP disposition was best described by a two-compartment model with first-order elimination. First-order kinetics described the absorption process after IM administration as occurred after oral administration [32].

Once given intravenously, CP rapidly achieved the pseudo-steady-state equilibrium, with a very long terminal phase accounting for most of the area under the plasma concentration–time profile. Among all the animal species in which the CP pharmacokinetics has been studied, swine shows the longest half-life values (t_1/2__λz_ IV = 36.34 ± 7.88 h, t_1/2__λz_ IM = 29.73 ± 5.56 h) [32] followed by humans [27] (t_1/2__λz_ IV = 9.91 ± 0.79 h, t_1/2__λzpo_ = 10.97 ± 1.40 h, t_1/2__λzpo_ = 16.01 ± 2.82 h) and horses [19] (11.35 ± 2.21 h).

Half-life values of our study were longer than those reported by Bošnjak M. et al. [32], after IV administration at the same dose (t_1/2__β_ = 17.31 h). These differences could be due to the sampling schedules applied during the terminal phase, to which non-compartmental analysis is very sensitive. The same reason could account for differences found between half-life values after IV and IM administrations in our study. The long half-life was rather attributed to a slow clearance than to an extensive distribution. CP is mainly eliminated by hepatic metabolism, though the metabolic pathway in pigs is still not known. According to the apparent half-life value observed after intramuscular administration, about 12 days are required for total CP removal from the body. Then, a minimal wash-out period of 15 days would be appropriate before human consumption of CP-treated pork.

The estimated clearance value (0.255 L/h or 0.0081 L/h/kg for a mean bodyweight of 31.5 kg) was much lower than plasma hepatic flow (3.12 L/kg/h) [48], confirming the restrictive clearance characteristics of CP as in other animal species.

In line with other animal species [18,19,20,21,22,23,24,25,26,27,28,29,30,31], the apparent distribution volume (Vss = Vc + VP = 9.62 L or 0.306 L/kg for a mean bodyweight of 31.5 kg) was lower than the total body water (0.63 L/kg) [48], due to the fact that CP is mainly confined in plasma according to its high protein binding as other NSAIDs [49].

The comparison of distribution volumes and clearance values among different species (Table S1 Supplementary Material) suggested that differences between half-life values observed in some cases are rather due to clearance than to distribution volume. As an example, differences in clearance values between swine (0.0081 L/h/kg for a mean bodyweight of 31.5 kg) and humans (0.329 L/kg [27]), are larger than between distribution volumes (0.306 L/kg for a mean bodyweight of 31.5 kg and 0.329 L/kg [27], for swine and humans, respectively).

Indeed, although differences in distribution volume among species exist, these are lower than differences in clearance values (Table S1 Supplementary Material). This suggests a common distribution pattern between all the species, subject to physicochemical characteristics rather than to physiological differences. The variation in metabolic pathways from one species to another could explain the larger observed differences in CL [50].

The high IM CP bioavailability found in our study (90.8%) was anticipated, provided that by this route, the first-pass effect through the liver is avoided. In any case, high oral bioavailability should also be expected, given the low extraction ratio characteristics of this drug.

The relative rapid absorption rate (Ka = 0.471 h^−1^, t_1_/2absorption = 1.47 h) observed after IM administration is in accordance with its high permeability as a class II drug of the biopharmaceutic classification system owing to its high lipophilicity. The absorption rate constant was in agreement with relatively short Tmax values found in the non-compartmental analysis.

Tissue concentrations (liver and kidney) at 48 h post-IM administration were about ten times lower than those found in plasma (3.27 µg/mL). The relative liver (0.19 µg/mL) and kidney (0.29 µg/mL) concentrations suggest that tissue/plasma partition coefficients are not very different between these tissues. According to the physiological tissue plasma flows in swine (liver: 52 +/− 6 and kidneys 15 +/− 2: mL/kg bodyweight /min) [51], both organs are well perfused so that perfusion should not be the rate-limiting factor of the CP tissue uptake.

The added value of PK models is that they can be used to predict plasma concentrations after different single doses or multiple-dose regimens once the therapeutic window has been established. Simulations showed that after IM administration of 4 mg/kg every 24 h, CP plasma concentrations would be similar to those achieved in humans and dogs, that in turn are considered therapeutic. In cases in which IV administration would be performed, 3 mg/kg every 24 h or 1.5 mg/kg every 12 h, would be appropriate. These results are in agreement with the dose administered to 38 patients by Furst et al. [12] in a clinical trial, in which they received from 100 to 800 mg/day. The authors observed adverse effects from 600 mg/day up, while lower doses of 100 and 300 mg/day were safe. In another clinical trial, CP was investigated in 50 patients with coxarthrosis at the dose of 300 mg/daily, obtaining good efficacy and showing few side-effects [14]. Jalava and co-workers also assessed the efficacy of 300 mg/day of CP in rheumatoid arthritis [13]. Therefore, the pig may be useful as animal model for in vivo evaluation of new CP formulations and dosage regimens as to perform pharmacokinetic-pharmacodynamic analysis before CP administration in humans.

Finally, the limitations of this study are the low number of animals included that in turn prevented inclusion of bodyweight on the distribution and flow pharmacokinetic parameters, allometrically, as occurred before [24]. Such a model could be useful to further predict the CP PK behavior in humans. Further studies will be required with a higher number of animals and more intensive tissue sampling to develop a physiological based pharmacokinetic model to predict human PK behavior.

## 5. Conclusions

We have characterized the disposition and IM bioavailability of CP in anesthetized swine as an animal model to study human anti-inflammatory diseases. Through a population pharmacokinetic approach, a similar distribution profile in pigs compared to other mammals has been shown. Otherwise, lower clearance values were found that led to the longest half-life among all the studied animal species. Differences between clearance values could be probably due to different metabolic pathways still pending investigations in swine.

CP remains unchanged in the organism for a longer period than in other mammals. Thus, it can exercise more lasting anti-inflammatory action. Hence, we can say that CP is a good and safe candidate to treat inflammatory diseases and pain in swine species. These results open the possibility of using Rimadyl^®^ for veterinary applications in this species and propose the swine as a good animal model to study other CP formulations and the pharmacokinetic–pharmacodynamic relationships before being used on humans.

## Figures and Tables

**Table 1:** Repeatability of the method for two transitions (T_1_ and T_2_) expressed as mean, standard deviation (SD) and relative standard deviation (RSD), for three different concentration levels (10, 250 and 750 ppb).

	T_1_			T_2_		
Units (ppb)	10	250	750	10	250	750
Mean	9180.11	161,6303.90	463,8409.44	2223.12	398,172.36	115,8660.25
SD	161.62	18078.15	51578.04	41.39	1613.87	9652.78
RSD	1.76	1.12	1.11	1.86	0.41	0.83

**Table 2:** Accuracy of the method for transitions T_1_ and T_2_ expressed by the mean, standard deviation (SD) and relative standard deviation (RSD), for seven concentration levels of standard curves, ranging from 1 ppb (ng/mL) to 1000 ppb.

	T_1_			T_2_		
Sample Name	Mean	SD	RSD	Mean	SD	RSD
RD_ppb_1	98.20	9.37	9.55	98.58	8.01	8.13
RD_ppb_10	117.75	2.87	2.44	114.75	2.87	2.50
RD_ppb_100	96.38	0.92	0.96	94.15	1.28	1.36
RD_ppb_250	103.25	1.50	1.45	102.50	1.73	1.69
RD_ppb_500	95.28	1.47	1.54	95.43	2.07	2.17
RD_ppb_750	94.33	2.96	3.14	96.08	4.36	4.54
RD_ppb_1000	94.70	1.66	1.75	98.80	2.31	2.33

**Table 3:** Median (min, max) values of CP concentrations remaining in liver and kidney at 48 h post intramuscular administration (4 mg/kg), expressed as CP µg per g of tissue.

Tissue	Concentration (µg/g) ^1^
Plasma	3.26 (3.21–3.60)
Liver	0.19 (0.001–0.37)
Kidney	0.29 (0.22–0.37)

^1^ Once the distribution equilibrium was reached, the highest concentrations occurred in plasma followed by kidney and liver.

**Table 4:** Mean ± SD (standard deviation) values of the main pharmacokinetic parameters estimated by the non-compartmental approach, after intravenous and intramuscular administration of 4 mg/kg CP to swine.

Parameter	Intravenous Administration	Intramuscular Administration
λz (h^−1^)	0.019 ± 0.005	0.024 ± 0.005
t_1/2λz_ (h)	36.34 ± 7.88	29.73 ± 5.56
AUC (µg/mL)·h	620.07 ± 168.10	403.57 ± 16.33
AUC_extrap_ (%)	62.35 ± 7.07	33.58 ± 7.22
CL (mL/h/kg)	6.86 ± 2.01	-
Vi (L/kg)	0.265 ± 0.015	-
Vss (L/kg)	0.342 ± 0.027	-
Vdarea (L/kg)	0.342 ± 0.027	-
Cmax (µg/mL)	15.11 ± 0.86	10.14 ± 2.11
Tmax (h)	-	4.0 (4.0–5.0)
F (%)	-	69.34 ± 21.00

λz—apparent elimination rate constant; t_1/2λz_—elimination half-life; AUC—area under the concentration vs. time curve; AUC_extrap_—percentage of the extrapolated area; CL—plasma clearance; Vi—initial distribution volume; Vdarea—distribution volume associated with the terminal phase; Vss—distribution volume at steady state; Cmax; peak concentrations; Tmax; time to peak concentration after intramuscular administration (median and range); F—bioavailability after IM administration, estimated as the ratio of dose-normalized AUC values after IM administration to dose-normalized AUC values after IV administration.

**Table 5:** Mean (relative standard errors, RSE%) values of the disposition and absorption pharmacokinetic parameters estimated by the final model.

Parameter		Units	Final Model Parameter Estimate (RSE%)
Disposition parameters	CL	L/h	0.255 (20.75)
	V_C_	L	7.11 (25.60)
	CL_D_	L/h	127.00 (21.50)
	V_P_	L	2.51 (92.43)
Release/Absorption parameters	F	%	90.80 (11.89)
	Ka	h^−1^	0.471 (49.26)
Inter-individual variability	IIV_CL_	%	33.17 (110.00)
	IIV_Vc_	%	33.47 (220.00)
	IIV_Ka_	%	95.86 (54.00)
Residual variability	Proportional	%	24.23 (21.47)

CL—plasma clearance; V_C_ and V_P_—volumes of distribution for central and peripheral compartments; CL_D_—intercompartmental clearance between central and peripheral compartments; IIV and residual variability given as coefficient of variation (%). Ka—first-order kinetics absorption rate constant; F—Bioavailability.

## Data Availability

The data presented in this study are available on request from the corresponding author.

## Supplementary Materials

The following supporting information can be downloaded at: https://www.mdpi.com/article/10.3390/pharmaceutics14051045/s1, Figure S1: Ionograms of blank samples with internal standard (IS, Carprofen-d3). (a) blank sample ionogram of swine 1; (b) blank sample ionogram of swine 2; (c) blank sample ionogram of swine 3 and (d) blank sample ionogram of swine 4; Figure S2: Ionograms of CP at different plasma samples times of swine number 1; (a) standard curve at 750 ng/mL; (b) intravenous levels at 5 min; (c) intravenous levels at 6 h; (d) intravenous levels at 24 h; (e) intramuscular levels at 5 min; (f) intramuscular levels at 6 h, and (g) intramuscular levels at 24; Figure S3: Ionograms of CP at different plasma samples times of swine number 2. A: IV levels at 5 min; B: IV levels at 6 h; C: IV levels at 24 h; D: IM levels at 5 min; E: IM levels at 6 h; F: IM levels at 24 h; Figure S4: Ionograms of CP at different plasma samples times of swine number 3. A: IV levels at 5 min; B: IV levels at 6 h; C: IV levels at 24 h; D: IM levels at 5 min; E: IM levels at 6 h; F: IM levels at 24 h; Figure S5: Ionograms of CP at different plasma samples times of swine number 4. A: IV levels at 5 min; B: IV levels at 6 h; C: IV levels at 24 h; D: IM levels at 5 min; E: IM levels at 6 h; F: IM levels at 24 h; Figure S6: Ionograms of Limit of Detection (LOD) and Limit of Quantification (LOQ) for: (a) transition T_1_, and (b) transition T_2_; Figure S7: Ionograms of determination of Specificity with blank samples of plasma without IS for: (a) transition T1 and (b) transition T2. Figure S8: Ionograms of Std (1 ppb and 10 ppb with IS 100 ppb), and samples with (100 ppb) and without IS: (a) Std 1ppb: Carprofen, (b) Std 1 ppb: IS (c) Std 10 ppb: Carprofen, (d) Std 10 ppb: IS, (e) Blank sample T0 with IS (100 ppb): carprofen, (f): Blank sample T0 with IS (100 ppb): IS, (g) Blank sample without IS: carprofen (h) Blank Sample without IS: IS signal. Figure S9: Goodness-of-fit plots for the final population pharmacokinetic model. Observed concentrations versus population predicted and individual predicted concentrations. Individual weighted residuals (IWRES) vs. individual predicted concentrations; conditional weighted residuals (CWRES) vs. time. Black line: identity line; red line: smooth line indicating the general data trend. Concentrations expressed as µg/mL, time given in hours. Table S1: CP pharmacokinetic parameter values in different animal species. Statistical comparison of the CP pharmacokinetic parameters (HLβ, AUC, Vdss, Cl and MRT) of swine versus different studied species such as: dogs (4 mg/kg IV Rimadyl^®^) [29], horses (4 mg/kg IV Rimadyl^®^) [19], cats (4 mg/kg IV Zenecarp^®^) [30], rabbits(2 mg/kg IV Rimadyl^®^) [24], sheep (4 mg/kg IV racemic CP) [23], humans (100 mg IV Imadyl^®^) [27], and the present study in swine (4 mg/kg IV Rimadyl^®^) expressed as mean ± SD, except for dogs and swine (mean and coefficient variation, given in parenthesis, when available).
