# Supplementary material for: Swine as the Animal Model for Testing New Formulations of Anti-Inflammatory Drugs: Carprofen Pharmacokinetics and Bioavailability of the Intramuscular Route
Source: Pharmaceutics. 2022 May 12;14(5):1045. doi: 10.3390/pharmaceutics14051045 (PMC9143053; doi:10.3390/pharmaceutics14051045)
Supplement: Supplementary file 1 [file pharmaceutics-14-01045-s001.zip › pharmaceutics-1682262-supplementary.pdf]

Supplementary material

Swine as the animal model for testing new formulations of anti-inflammatory drugs: Carprofen Pharmacokinetics and Bioavailability of the intra-muscular route

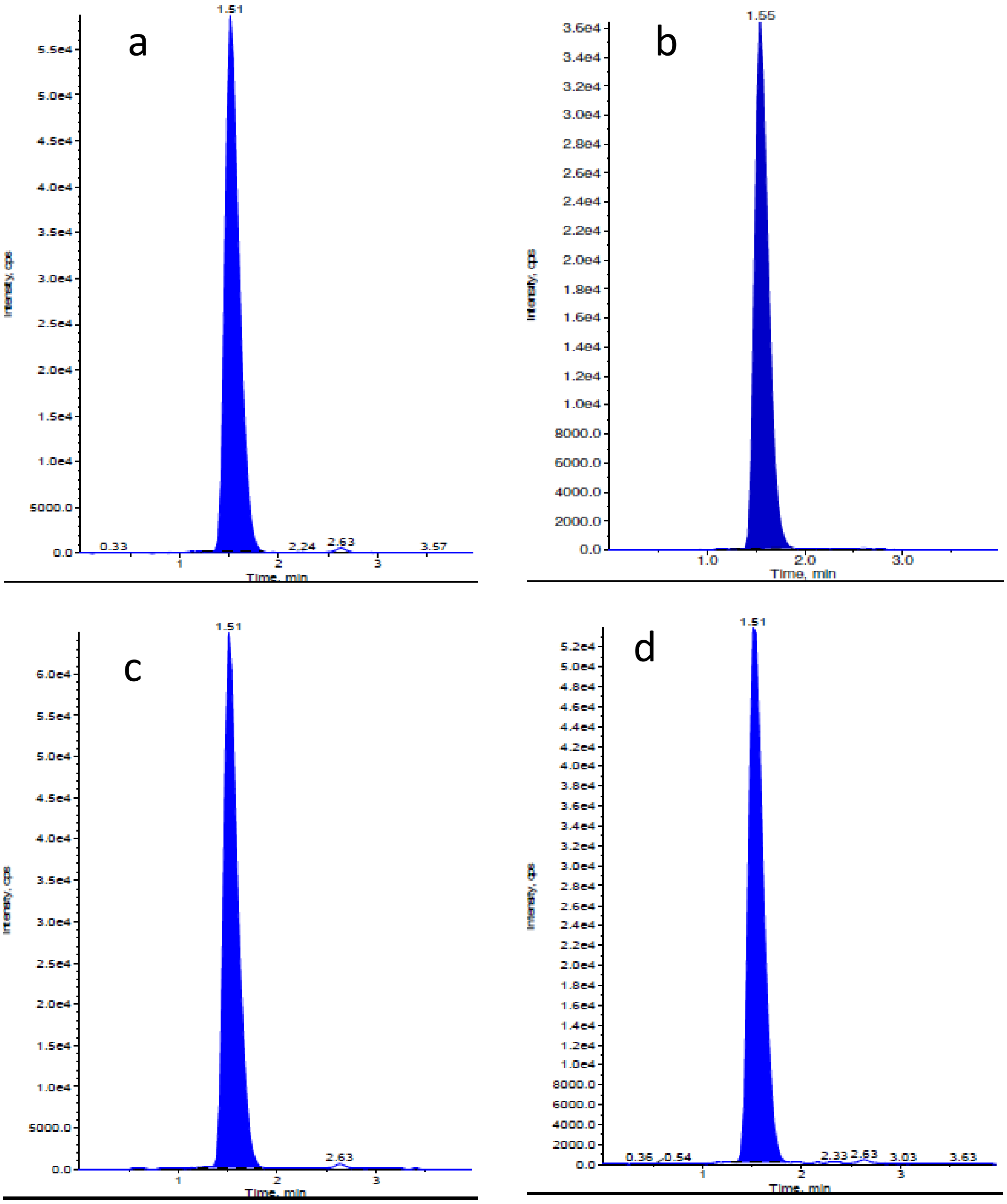

Figure S1. Ionograms of blank samples with internal standard (IS, Carprofen-d3). (a) blank sample ionogram of swine 1; (b) blank sample ionogram of swine 2; (c) blank sample ionogram of swine 3 and (d) blank sample ionogram of swine 4.

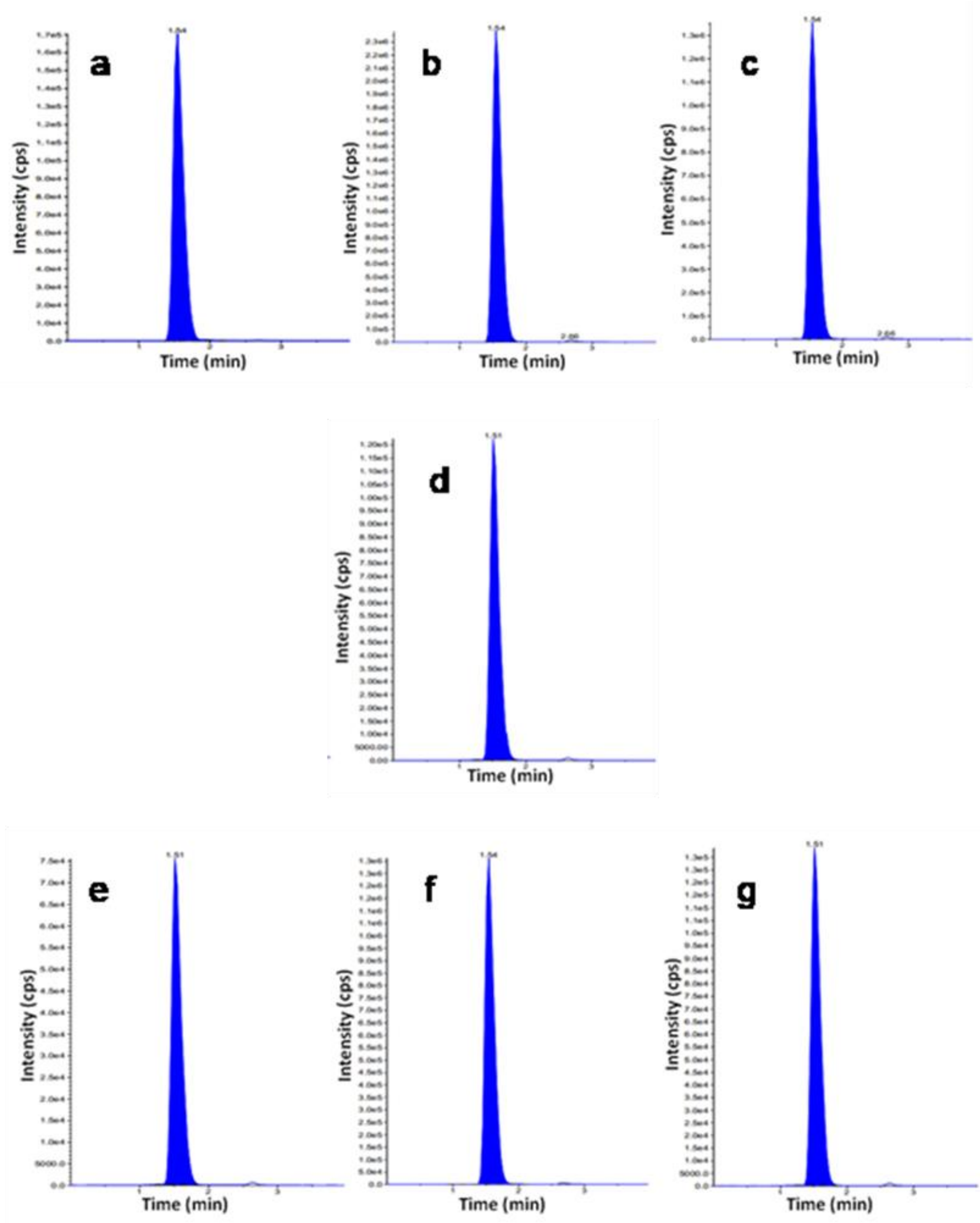

Figure S2. Ionograms of CP at different plasma samples times of swine number 1; (a) standard curve at 750 ng/mL; (b) intravenous levels at 5 min; (c) intravenous levels at 6 h; (d) intravenous levels at 24 h; (e) intramuscular levels at 5 min; (f) intramuscular levels at 6 h, and (g) intramuscular levels at 24 h.

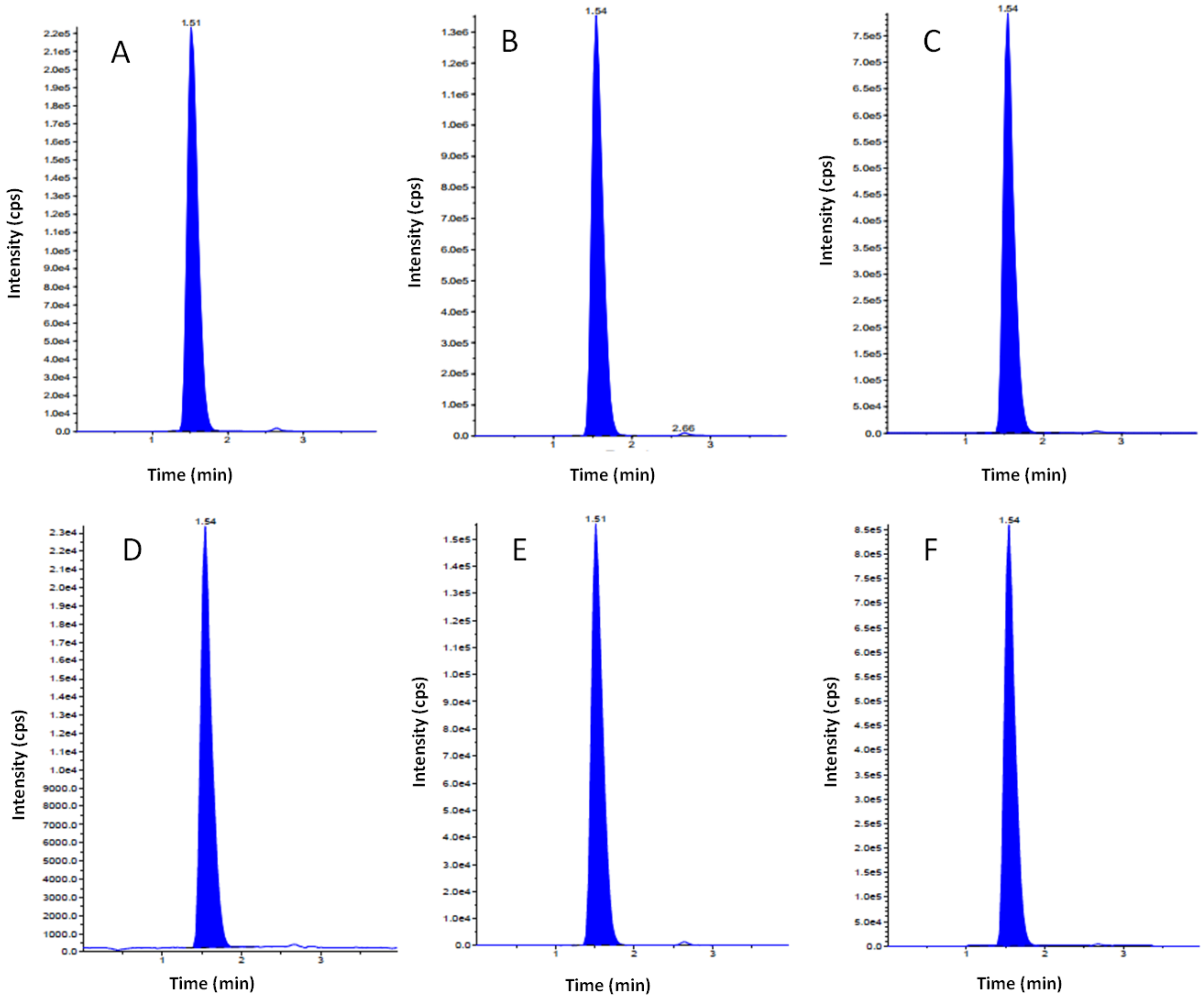

Figure S3: Ionograms of CP at different plasma samples times of swine number 2. A: IV levels at 5 min; B: IV levels at 6h; C: IV levels at 24h; D: IM levels at 5 min; E: IM levels at 6h and F: IM levels at 24h.

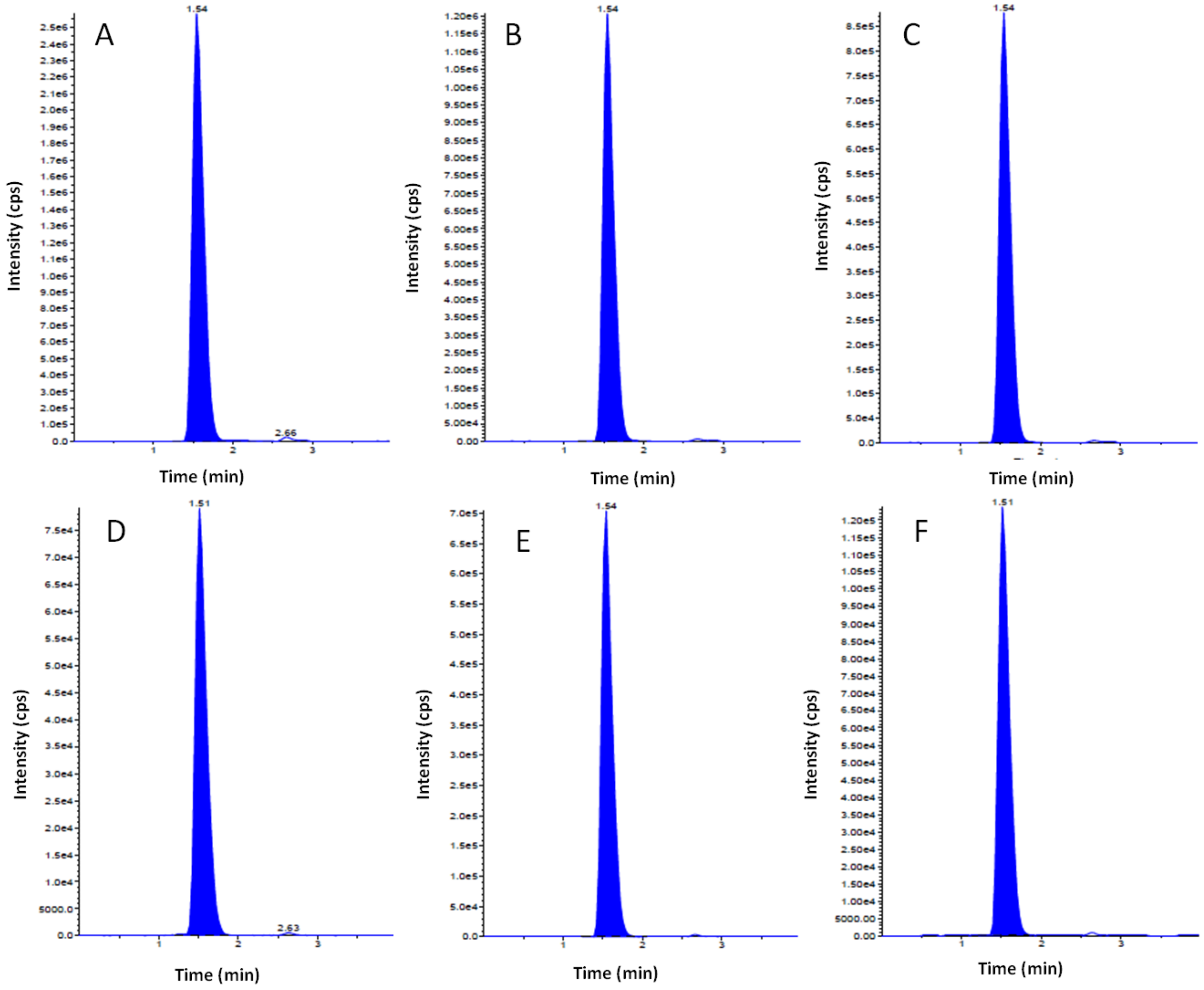

Figure S4: Ionograms of CP at different plasma samples times of swine number 3. A: IV levels at 5 min; B: IV levels at 6h; C: IV levels at 24h; D: IM levels at 5 min; E: IM levels at 6h and F: IM levels at 24h.

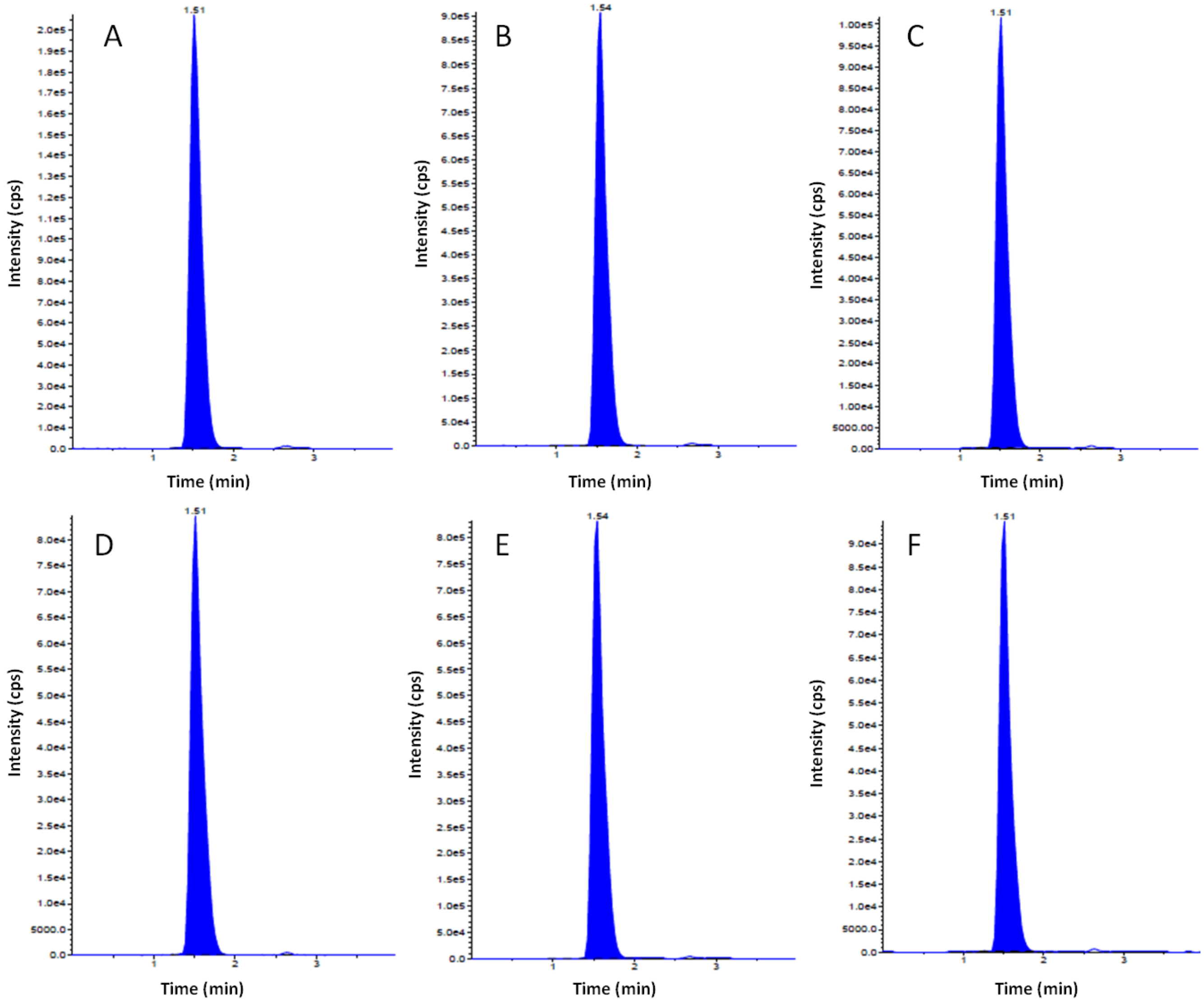

Figure S5: Ionograms of CP at different plasma samples times of swine number 4. A: IV levels at 5 min; B: IV levels at 6h; C: IV levels at 24h; D: IM levels at 5 min; E: IM levels at 6h and F: IM levels at 24h.

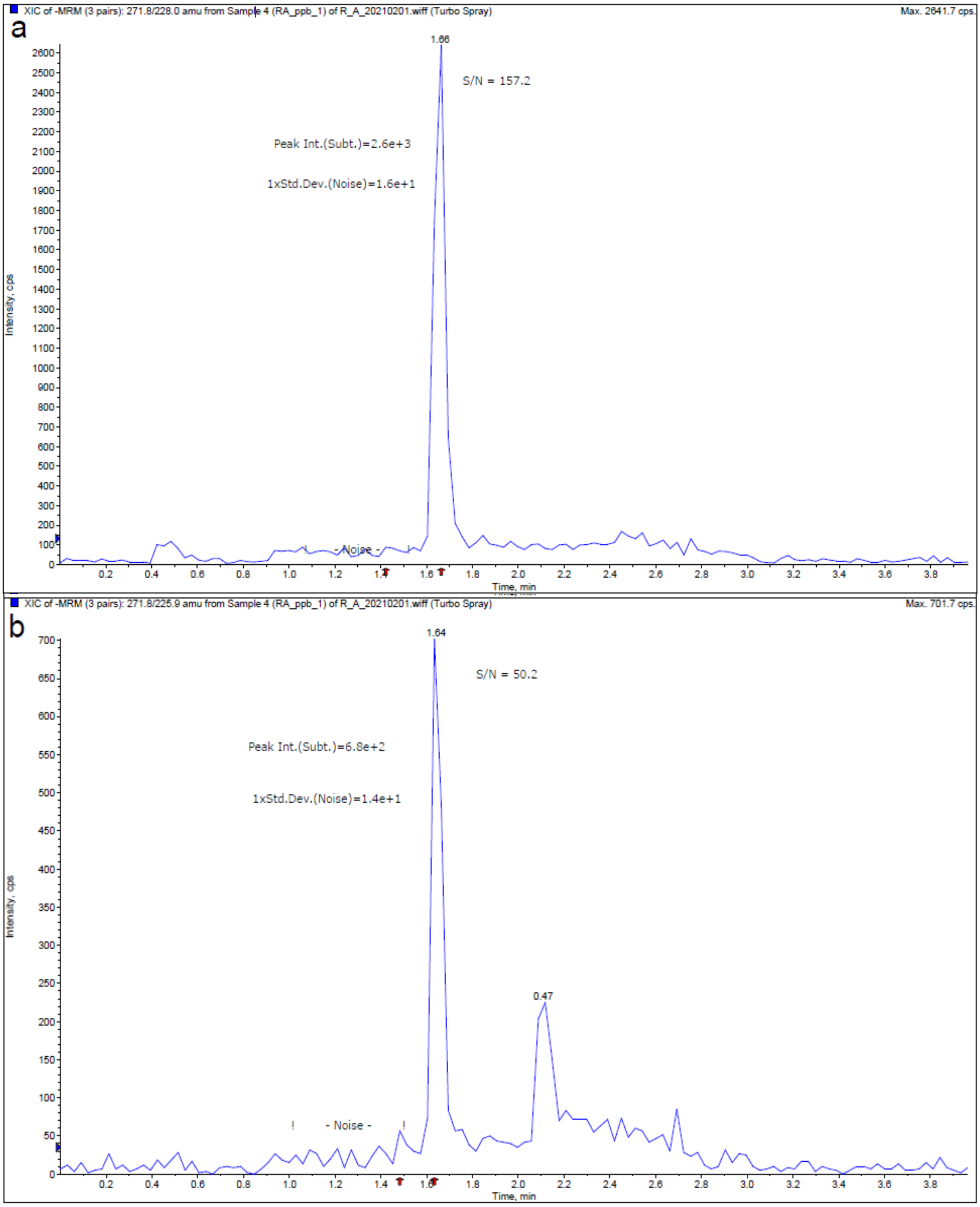

Figure S6: Ionograms of Limit of Detection (LOD) and Limit of Quantification (LOQ) for: (a) transition T1, and (b) transition T2.

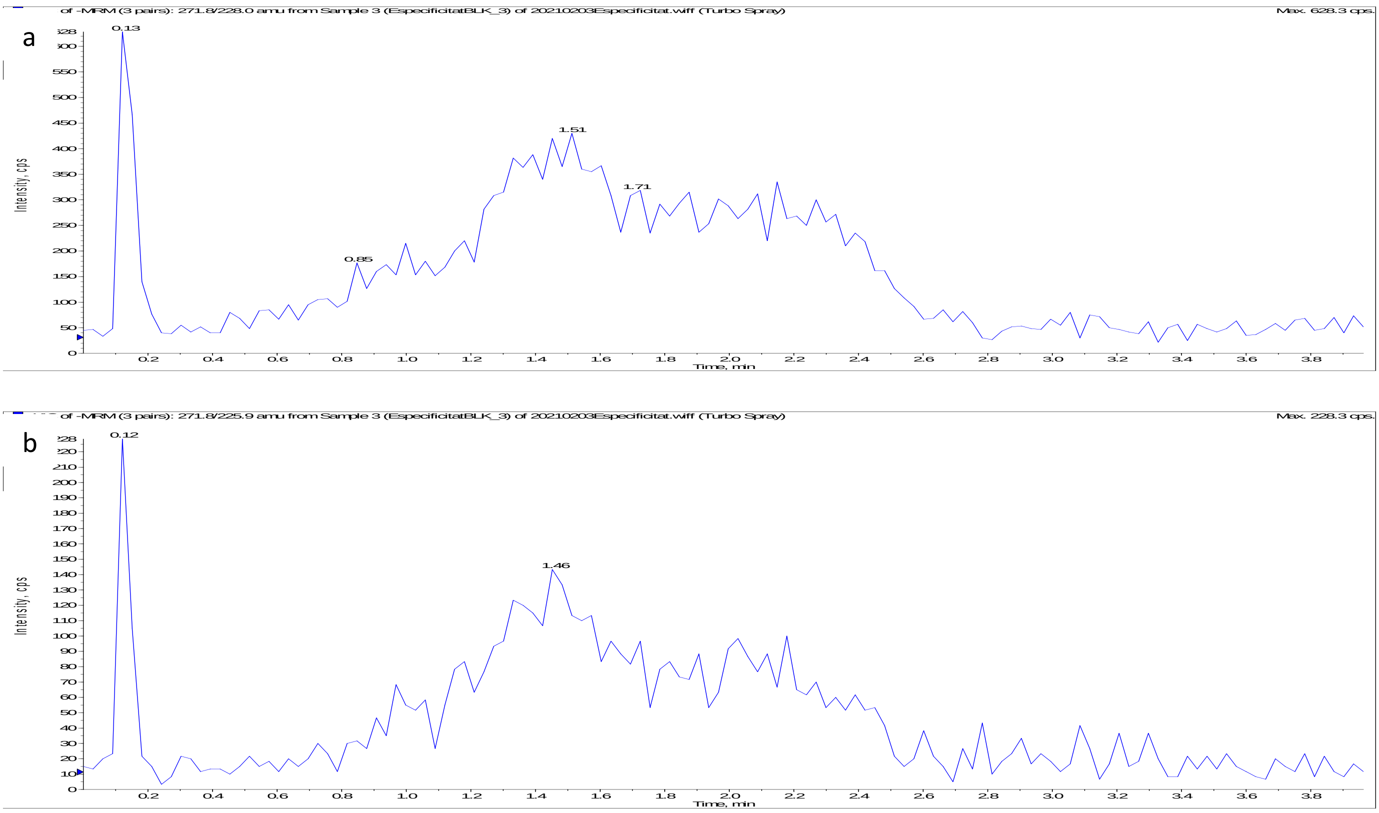

Figure S7: Ionograms of determination of Specificity with blank samples of plasma without IS for: (a) transition T1 and (b) transition T2.

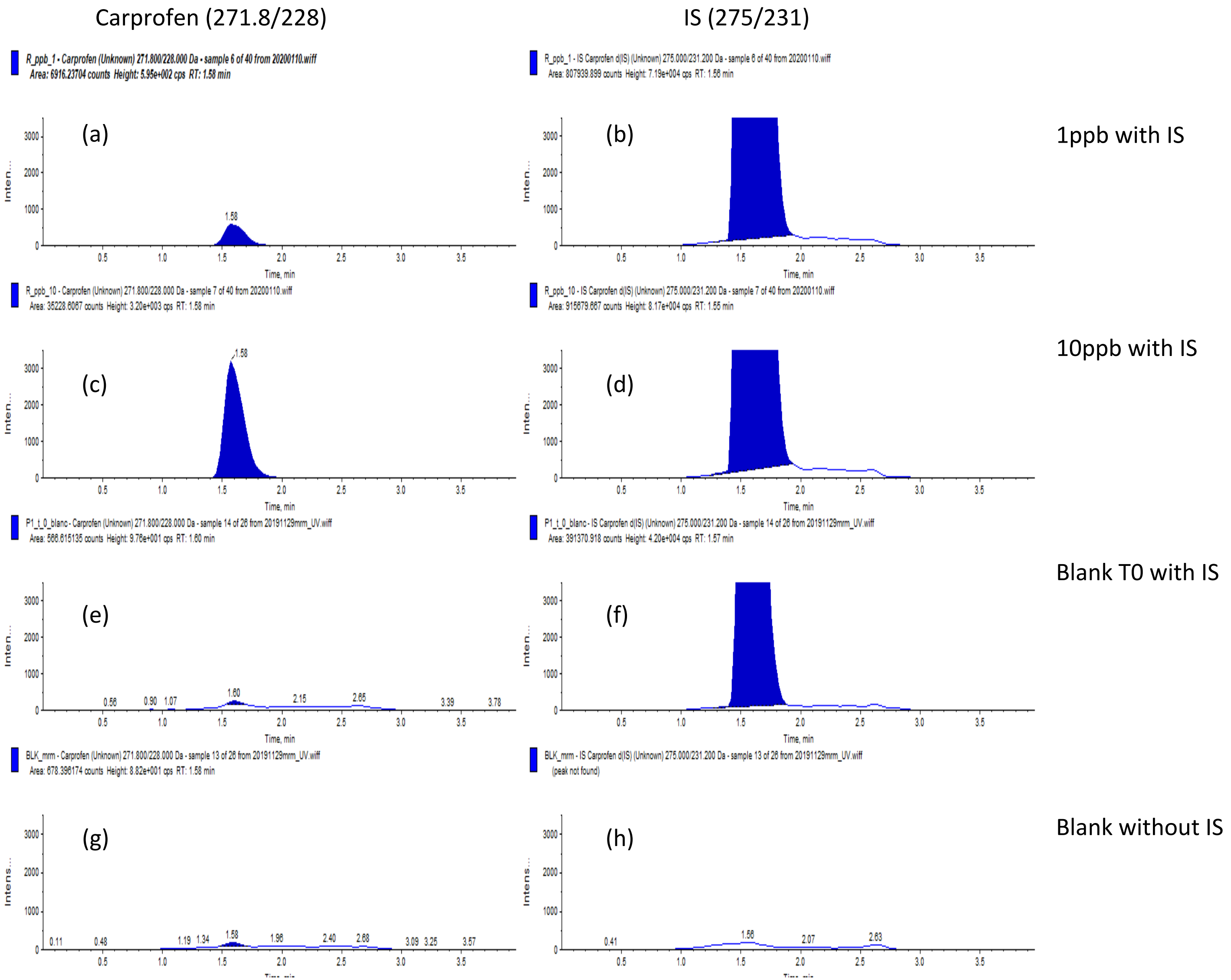

Figure S8: Ionograms of Std (1 ppb and 10 ppb with IS 100 ppb), and samples with (100 ppb) and without IS: (a) Std 1ppb: Carprofen, (b) Std 1 ppb: IS (c) Std 10 ppb: Carprofen, (d) Std 10 ppb: IS, (e) Blank sample T0 with IS (100 ppb): carprofen, (f): Blank sample T0 with IS (100 ppb): IS, (g) Blank sample without IS: carprofen (h) Blank Sample without IS: IS signal.

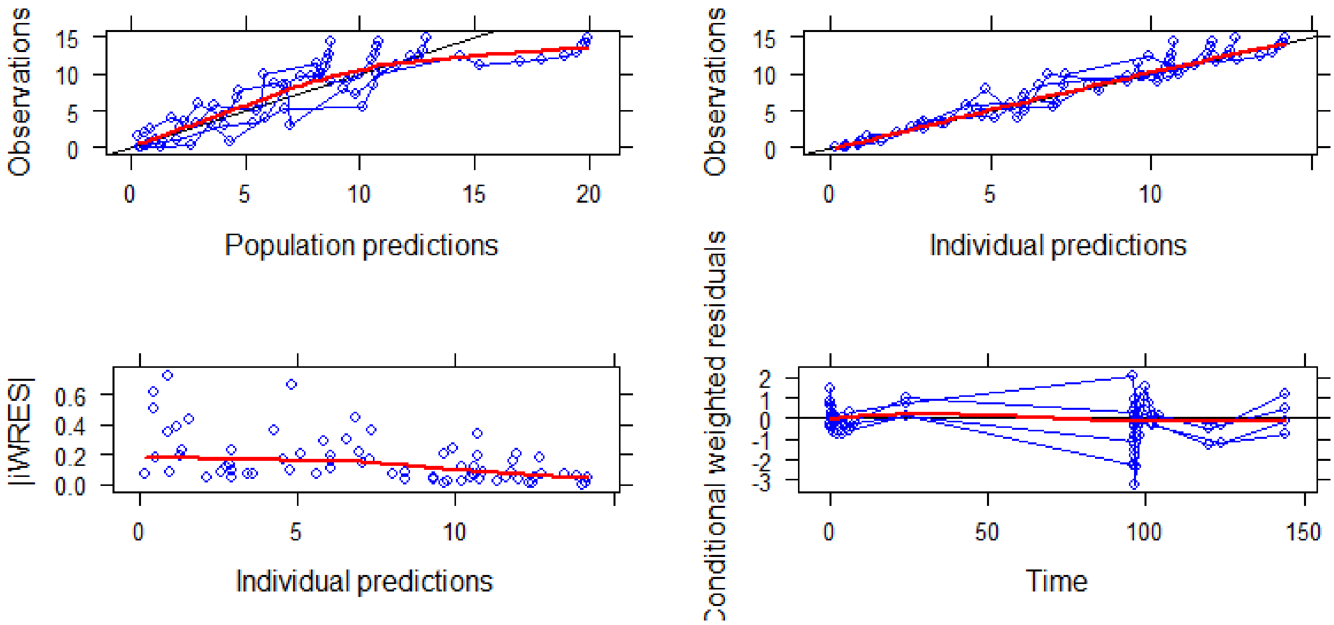

Figure S9. Goodness-of-fit plots for the final population pharmacokinetic model. Observed concentrations versus Population predicted and individual predicted concentrations. Individual weighted residuals (IWRES) vs individual predicted concentrations; Conditional weighted residuals (CWRES) vs time. Blackline: identity line; Red line: Smooth line indicating the general data trend. Concentrations expressed as μg/mL, Time given in hours.

Table S1: CP Pharmacokinetic parameter values in different animal species. Statistical comparison of the CP pharmacokinetic parameters (HLβ, AUC, Vdss, Cl and MRT) of swine versus different studied species such as: dogs (4 mg/kg IV Rimadyl®) [29], horses (4mg/kg IV Rimadyl®) [19], cats (4 mg/kg IV Zenecarp®) [30], rabbits (2 mg/kg IV Rimadyl®) [24], sheep (4 mg/kg IV racemic CP) [23], humans (100 mg IV Imadyl®) [27], and the present study in swine (4 mg/kg IV Rimadyl®) expressed as mean ± SD, except for dogs and swine (mean and coefficient variation, given in parenthesis, when available).

**Parameters**	**Dog (n=6)**	**Horse (n=6)**	**Cat (n=5)**	**Rabbit (n=6)**	**Sheep (n=8)**	**Human (n=6)**	**Swine (n=4)**
**Co** *(μg/mL)*	58.11 *(100.70)*	36.16 ± 3.88	179.80 ± 263.10	20.70 ± 2.05	39.61 ± 5.60	ND	-
**A** *(μg/mL)*	47.68 (13.39)	28.38 ± 3.26	146.00 ± 239.00	14.36 ± 2.72	ND	ND	-
**B** *(μg/mL)*	10.43 (87.31)	7.78 ± 0.61	33.80 ± 24.10	6.10 ± 2.08	ND	ND	-
**α** *(h^{-1})*	0.43 (25.13)	2.01 ± 0.66	2.94 ± 3.90	1.94 ± 0.66	1.69 ± 0.33	ND	-
**β** *(h^{-1})*	0.08 (17.15)	0.06 ± 0.01	0.05 ± 0.02	0.23 ± 0.05	0.03 ± 0.01	ND	-
**$t_{1/2}$α** *(h)*	1.63 (25.13)	0.39 ± 0.13	ND	0.36 ± harmonic	0.43 ± 0.09	1.80 ± 0.50	-
**$t_{1/2}$β** *(h)*	9.10 (17.15)	11.35 ± 2.21 *	20.00 ± 16.60	3.06 ± harmonic	24.17 ± 5.98	9.91 ± 0.79 *	36.34 *(21.68)*$^{\$}$
**AUC** *(μg · h/mL)*	268.94 (26.00)***	138.1 ± 17.89 ***	636.00 ± 237.00	37.82 ± 9.64 ***	531.80 ± 120.70	ND	-
**K_{10}** *(h^{-1})*	0.23 (26.61)	0.22 ± 0.04	0.34 ± 0.53	0.55 ± 0.21	0.08 ± 0.04	ND	-
**K_{12}** *(h^{-1})*	0.11 (61.30)	1.27 ± 0.49	2.10 ± 3.3	0.81 ± 0.34	0.97 ± 0.24	ND	-
**K_{21}** *(h^{-1})*	0.14 (43.31)	0.59 ± 0.20	0.55 ± 0.55	0.81 ± 0.16	0.68 ± 0.06	ND	-
**Vc** *(L/kg)*	0.07 (0.06)	0.14 ± 0.02	0.05 ± 0.03	0.10 ± 0.01	0.10 ± 0.00	0.045 ± 0.01	0.23 *(25.60)*$^{\&}$
**Vss** *(L/kg)*	0.12 (0.01) ***	0.43 ± 0.05 *	0.14 ± 0.05 ***	0.09 ± 0.02 ***	0.13 ± 0.19 *	0.22 ± 0.06 **	0.31$^{\&}$
**CL** *(mL/kg/h)*	14.87 (26.03)	29.00 ± 4.90 ***	7.10 ± 2.81	27.92 ± 7.07 ***	4.00 ± 1.22 *	31.11 ± 6.00 ***	8.17 *(20.75)*$^{\&}$
**MRT** *(h)*	8.02 (23.28) **	15.10 ± 3.43 ***	23.60 ± 16.2 *	3.45 ± 0.5 ***	33.89 ± 8.38 **	7.07 ± 9.17 ***	-

* P-Value <0.05; **P-Value <0.01 and ***P-Value <0.001; ND: Not determined**;** $: estimated form the non-compartmental approach; &: estimated from the population pharmacokinetic approach

Co: Initial concentration after iv bolus administration; A and B, α, β: Ordinate intercept terms and rate constants of the distribution and elimination phases, respectively; AUC: area under the curve of plasma concentrations vs time; K10, elimination rate constant from the central compartment; K12 and K21: distribution rate constants from and to the central compartment, respectively; Vc: central compartment distribution volume; Vss: steady-state distribution volume; CL: plasma clearance; MRT: mean residence time.
